# PRAME Staining of Adnexal Lesions and Common Skin Cancer Types: Biomarker with Potential Diagnostic Utility

**DOI:** 10.3390/dermatopathology11040039

**Published:** 2024-12-12

**Authors:** Hisham F. Bahmad, John Alexis

**Affiliations:** 1 Arkadi M. Rywlin M.D. Department of Pathology and Laboratory Medicine, Mount Sinai Medical Center, Miami Beach, FL 33140, USA; john.alexis@msmc.com; 2 Department of Pathology, Herbert Wertheim College of Medicine, Florida International University, Miami, FL 33199, USA

**Keywords:** PRAME, adnexal lesions, sebaceous, skin cancer, biomarker

## Abstract

PRAME (PReferentially expressed Antigen in MElanoma) is a tumor-associated antigen first identified in tumor-reactive T-cell clones derived from a patient with metastatic melanoma. Immunohistochemistry (IHC) for PRAME is useful for diagnostic purposes to support a suspected diagnosis of melanoma. Anecdotally, PRAME has been observed to stain sebaceous units in glands in background skin. We examined the expression of PRAME in adnexal lesions and common skin cancers to determine whether it is of potential diagnostic utility in supporting the differentiation between sebaceous and non-sebaceous lesions. IRB approval from Mount Sinai Medical Center (MSMC) was obtained. This is a single-center retrospective cohort analysis over a ten-year period (1 January 2012, and 31 December 2023). We used the pathological database of skin lesions, including sebaceous, sweat gland, and follicular lesions, in addition to basal cell carcinomas (BCCs) and squamous cell carcinomas (SCCs), from 81 patients who underwent shave/punch biopsies or surgical excisions. We evaluated the IHC staining percentage positivity and intensity for PRAME. Staining intensity was subcategorized into negative, weak, moderate, and strong, whereas expression percentage positivity was subcategorized into 0%, 1–25%, 26–50%, 51–75%, and 76–100%. Most sebaceous versus non-sebaceous lesions exhibited cytoplasmic staining of moderate to strong intensity in >75% of cells. PRAME has a sensitivity and specificity of 100.0% and 86.7%, respectively, to support distinguishing between sebaceous and non-sebaceous adnexal lesions (regardless of whether they are benign or malignant). BCCs and SCCs showed weak to moderate nuclear staining for PRAME in >75% of cells. None of the 13 lesions of hair follicle origin showed any staining. A total of 26 of the 32 lesions of sweat gland origin were negative while 6 (18.75%) showed positive staining. In conclusion, we confirm the potential utility of PRAME for supporting the distinction between sebaceous and non-sebaceous adnexal lesions on one hand, and on the other, distinguishing BCC and SCC that may show nuclear staining from sebaceous carcinoma that shows cytoplasmic staining.

## 1. Introduction

Cutaneous neoplasms encompass a diverse array of tumors arising from various components of the skin, including the epidermis, adnexal structures, and dermis. Accurate diagnosis and classification of these lesions are essential for guiding appropriate management and prognostication. While traditional histopathological examination and morphology remains the cornerstone of diagnosis, ancillary techniques such as immunohistochemistry (IHC) have emerged as valuable adjuncts, facilitating the characterization of tumors, especially when biopsies contain only a small portion of the lesion.

One such marker that has garnered increasing attention in the realm of cutaneous pathology is PRAME (PReferentially expressed Antigen in MElanoma) [1]. Originally identified as a tumor-associated antigen in melanoma, PRAME has since been implicated in a spectrum of malignancies, including hematological and solid tumors such as non-small cell lung cancer, breast carcinoma, renal cell carcinoma, ovarian carcinoma, leukemia, synovial sarcoma, and myxoid liposarcoma [2,3,4,5,6,7,8]. In cutaneous pathology, PRAME expression has been predominantly studied in melanocytic lesions, where its detection by IHC has proven useful in supporting the diagnosis of melanoma [9].

Beyond its role in melanocytic tumors, emerging evidence suggests that PRAME may also play a diagnostic role in non-melanocytic cutaneous neoplasms, particularly those arising from adnexal structures [10]. Adnexal lesions encompass a heterogeneous group of tumors originating from the pilosebaceous unit and sweat glands. Distinguishing between different types of adnexal neoplasms can pose diagnostic challenges due to overlapping histological features and variable clinical presentations. Therefore, the identification of diagnostic markers that can support the distinction between different types of adnexal tumors is paramount in this context [10,11].

It is known that PRAME consistently stains sebaceous glands in normal skin. This finding prompted us to investigate staining for PRAME in adnexal lesions, including sebaceous, sweat gland, and follicular lesions. Furthermore, given the diverse array of cutaneous malignancies encountered in clinical practice, we sought to explore the potential utility of PRAME in common skin cancers, such as basal cell carcinoma (BCC) and squamous cell carcinoma (SCC).

In this study, we aimed to assess the potential utility of PRAME staining for supporting the differentiation between sebaceous and non-sebaceous adnexal lesions and elucidate its staining patterns in common skin cancers. We hypothesized that PRAME may serve as a valuable ancillary marker in the histopathological evaluation of cutaneous neoplasms, aiding in the accurate classification and subtyping of these tumors.

To achieve our objectives, we conducted a retrospective analysis of tissue samples obtained from patients with sebaceous, sweat gland, and follicular lesions, as well as BCC and SCC. IHC staining for PRAME was performed, and the staining patterns were correlated with histopathological features to delineate distinct expression patterns associated with different types of cutaneous neoplasms.

To the best of our knowledge, our study is amongst the first studies to elucidate the role of PRAME in adnexal cutaneous pathology, particularly spanning a broader spectrum of adnexal lesions and common skin cancers [9,10].

## 2. Materials and Methods

### 2.1. Study Design, Setting, and Objectives

This single-center retrospective cohort study was conducted following approval from the Institutional Review Board (IRB) of Mount Sinai Medical Center (MSMC). A comprehensive review of the pathological database at MSMC was performed to identify patients with cutaneous lesions diagnosed between 1 January 2012, and 31 December 2023. Inclusion criteria encompassed patients of all ages and genders with histopathologically confirmed cutaneous lesions, including adnexal lesions and common skin cancer-types. Objectives of our study included the following: (1) examining the immunohistochemical (IHC) protein staining of PRAME in cutaneous lesions, (2) exploring whether IHC could be valuable as adjunct information for supporting the distinction of sebaceous from non-sebaceous adnexal lesions, and lastly (3) comparing our clinical experience with data published in the literature in an attempt to uncover the diagnostic utility of PRAME in common skin cancer-types. This study represents analysis of patients treated at a large academic medical center. We reviewed the patients’ medical charts and collected patients’ information.

### 2.2. Ethical Considerations

Approval of the Institutional Review Board (IRB) of our academic medical center was granted prior to commencement of the study. All protocols followed in our retrospective cohort study were performed in accordance with guidelines and regulations of The Code of Ethics of the World Medical Association (Declaration of Helsinki). Chart review was carried out by Collaborative Institutional Training Initiative (CITI)-certified resident physicians and patients’ confidentiality was maintained. All data collected were de-identified and stored at the principal investigator’s office.

### 2.3. Patient Selection

Inclusion criteria included patients who underwent shave/punch biopsies or surgical excisions for skin lesions during the time period specified. We included only patients with unequivocal diagnoses and excluded patients who received neoadjuvant therapy prior to surgery or patients who had more than one distinct primary skin neoplasm.

In total, 81 patients were included:

- Sebaceous lesions (17 cases with prominent benign sebaceous glands, hyperplasia, heterotopia, adenoma, steatocystoma, epithelioma, or carcinoma)
- Sweat gland lesions (32 cases with hyperplasia, syringoma, hidradenoma, hidrocystoma, poroma, spiradenoma, papillary hidradenoma, or carcinoma)
- Follicular lesions (13 cases with trichoadenoma, pilomatricoma or pilomatrixoma, or trichilemmal cyst)
- Basal cell carcinoma (10 cases)
- Squamous cell carcinoma (9 cases)

### 2.4. Histology, Immunohistochemistry, and Evaluation of Staining

Formalin-fixed, paraffin-embedded (FFPE) tissue blocks corresponding to the selected cases were retrieved from the archives of the Department of Pathology at MSMC. Hematoxylin and eosin (H&E)-stained slides from each case were reviewed by a board-certified dermatopathologist (J.A.) to confirm the diagnosis and select representative tissue blocks for immunohistochemical analysis. IHC for PRAME was performed on 4 μm thick whole sections for each case on an automated Benchmark ULTRA staining platform (Ventana Medical Systems, Tucson, AZ, USA). The rabbit antibody was received prediluted and the antibody clone used was RBT-PRAME for PRAME (BioSB Bioscience for the World, Santa Barbara, CA, USA). A red-colored chromogenic stain was used in the Ventana Benchmark ULTRA automated slide staining system, where the “red” indicates the color of the staining reaction.

A dermatopathologist (J.A.) and a pathology resident (H.F.B.) reviewed the slides together and reached a consensus on the IHC staining percentage positivity and intensity. Staining intensity was subcategorized into negative, weak, moderate, and strong, as previously described [9]. Staining percentage positivity was subcategorized into 0%, 1–25%, 26–50%, 51–75%, and 76–100%. Different categories have been used in various studies to quantitatively assess the percentage of stained cells [12,13,14].

### 2.5. Statistical Analysis

Data retrieved from medical charts and pathology reports of patients were entered into a Microsoft Excel spreadsheet designed specifically for this study. The statistical analyses, data management, and cleaning were executed using Microsoft Excel. Descriptive statistics were reported as frequencies and percentages for categorical variables. The Chi-squared χ^2^ test was used to evaluate whether a significant association was present between the immunohistochemical staining (percentage and intensity) for PRAME. Sensitivities and specificities for PRAME staining were calculated. *p* values less than 0.05 were considered statistically significant.

## 3. Results

### 3.1. PRAME Staining in Sebaceous Lesions

**Figure 1:**
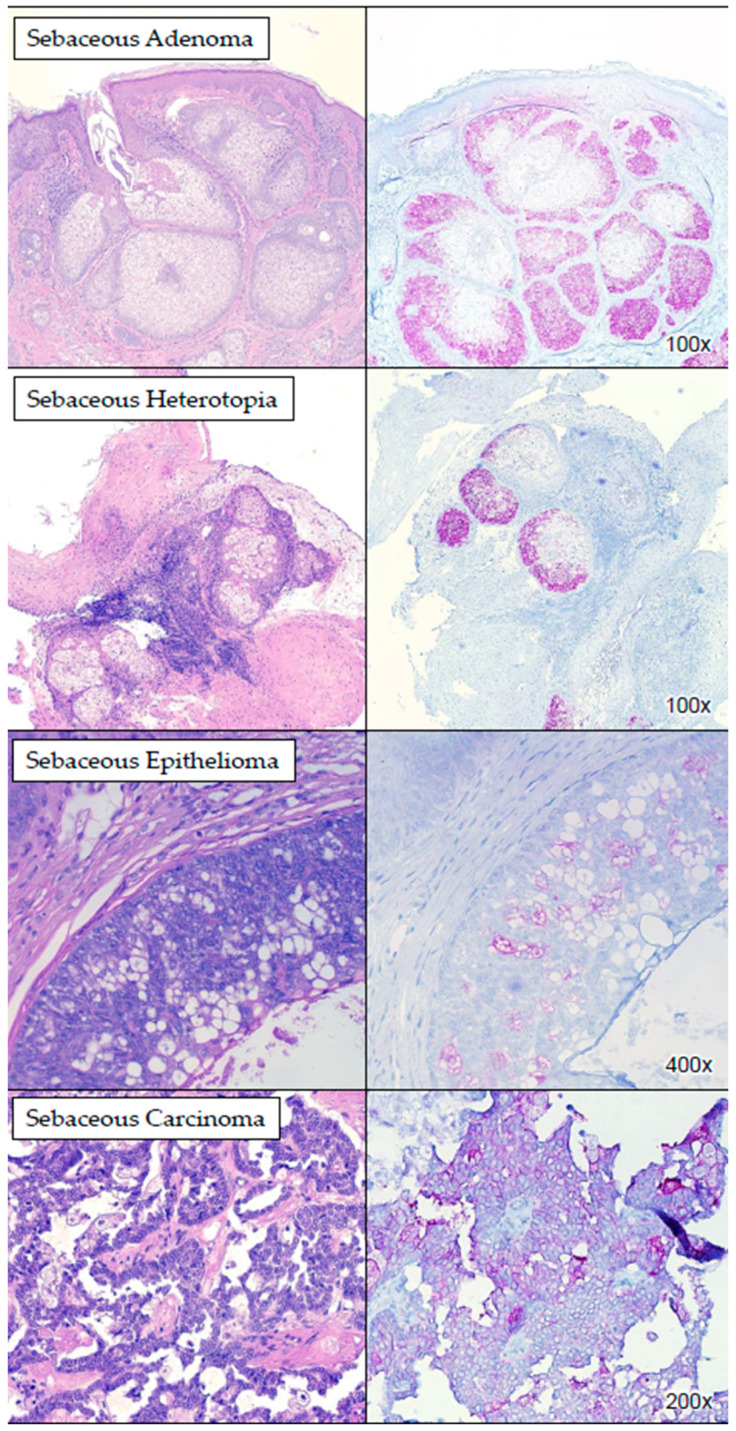
PRAME IHC staining in sebaceous lesions. The images show examples of benign and malignant sebaceous lesions which stained positively with PRAME (cytoplasmic staining).

Immunohistochemical analysis revealed positive staining with PRAME in the cytoplasm of sebaceous cells in all examined cases of sebaceous lesions (*n* = 17). The staining intensity ranged from moderate to strong, with >75% of cells demonstrating positivity (Table 1, Table 2 and Table 3). Notably, PRAME staining was predominantly peripheral within the sebaceous units, diminishing towards the center (Figure 1), which is most evident in well-differentiated sebaceous lesions. The sensitivity and specificity of PRAME for supporting the differentiation between sebaceous and non-sebaceous adnexal lesions were 100.0% and 86.7%, respectively. Among the different subtypes of sebaceous lesions, sebaceous carcinoma exhibited less PRAME staining. Supplementary Table S1 demonstrates the staining intensity and percentage of the different subtypes of sebaceous lesions. Most of the sebaceous lesions in our study were either benign entities or well-differentiated carcinomas. In the majority of those cases, PRAME picked up the well-formed sebocytes easily, which suggests that PRAME is very sensitive and specific for sebaceous lesions. In our cohort, we had one poorly differentiated carcinoma, and it showed a low percentage of PRAME expression as expected and as demonstrated in other studies.

### 3.2. PRAME Staining in Non-Sebaceous Adnexal Lesions

**Figure 2:**
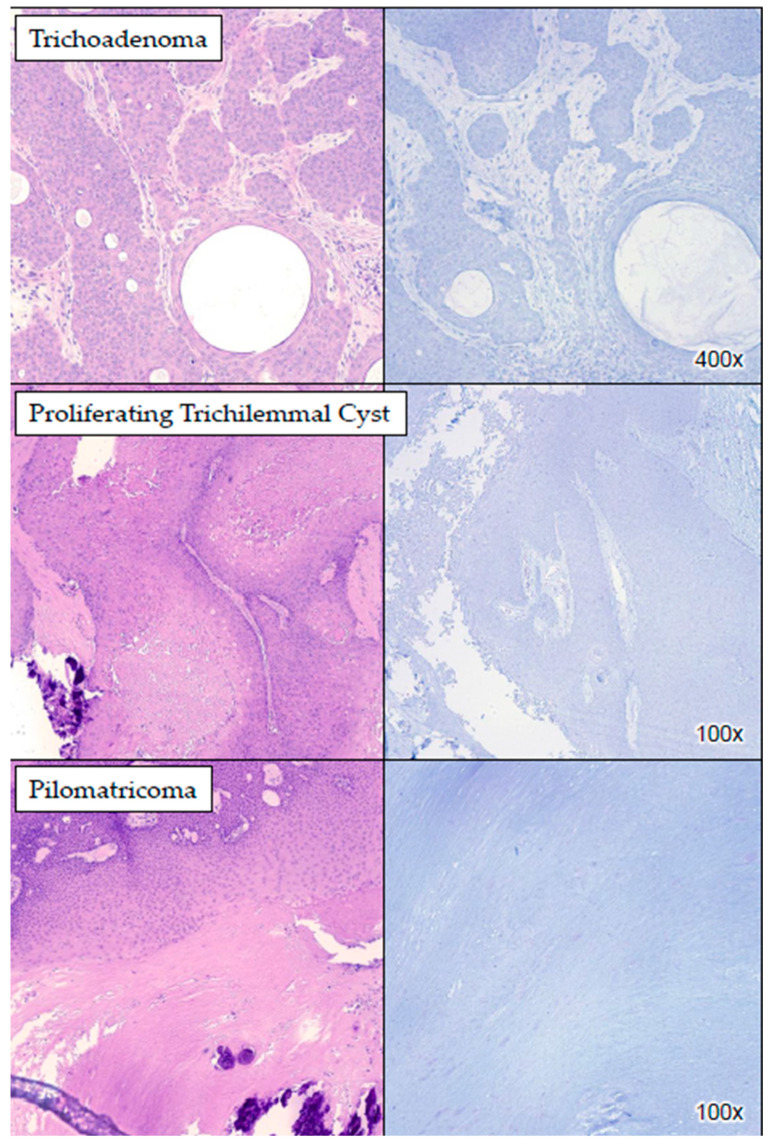
PRAME IHC staining in follicular lesions. The images show examples of follicular lesions which did not stain with PRAME.

**Figure 3:**
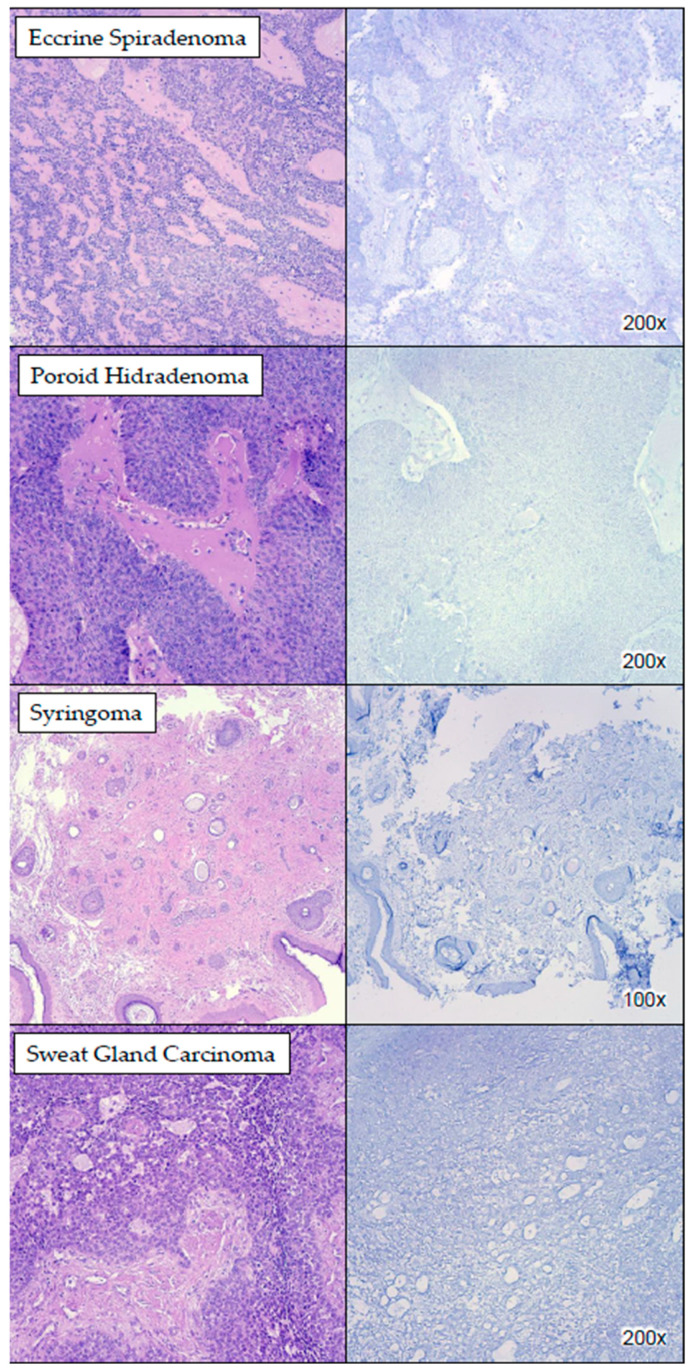
PRAME IHC staining in sweat gland lesions. The images show examples of sweat gland lesions which did not stain with PRAME.

None of the lesions of hair follicle origin (*n* = 13), which were all benign in our cohort, demonstrated PRAME positivity (Figure 2 and Supplementary Table S2). Of the 32 lesions of sweat gland origin, 26 (81.25%) did not stain with PRAME while 6 (18.75%) did (Figure 3). The six cases (18.75%) of sweat gland tumors that exhibited cytoplasmic PRAME staining were all benign, albeit with a lower percentage and intensity compared to sebaceous lesions. Supplementary Table S3 demonstrates the staining intensity and percentage of the different subtypes of sweat gland lesions.

### 3.3. PRAME Staining in Common Skin Cancers

**Figure 4:**
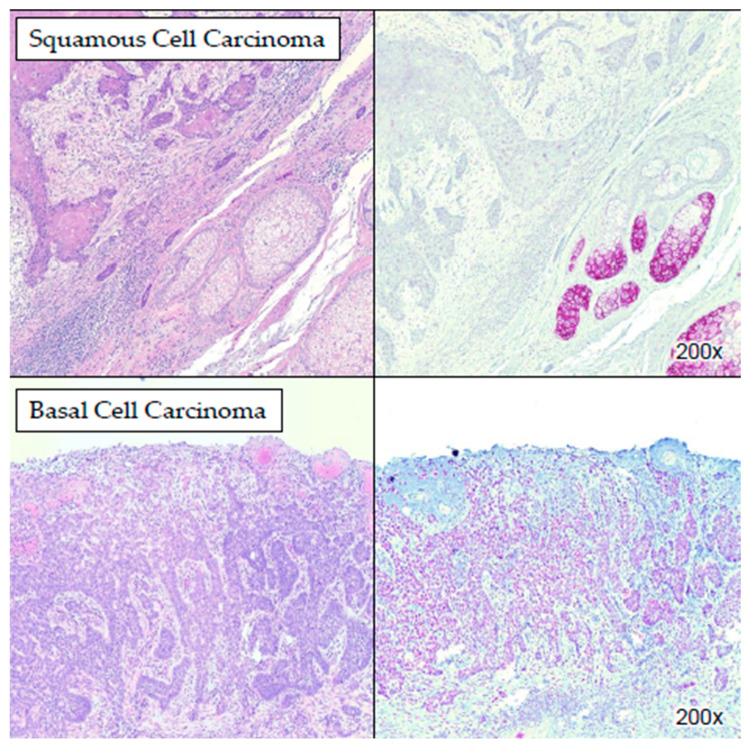
PRAME IHC staining in SCC and BCC. In the upper panel (squamous cell carcinoma), the neoplastic cells demonstrated weak nuclear staining for PRAME while the internal control sebaceous glands stained strongly positive for PRAME (cytoplasmic staining pattern). In the lower panel (basal cell carcinoma), the neoplastic cells demonstrated moderate nuclear staining for PRAME in >75% of cells.

Basal cell carcinomas (BCCs) and squamous cell carcinomas (SCCs) demonstrated weak to moderate nuclear staining for PRAME in >75% of cells (Figure 4). This nuclear staining pattern contrasted with the cytoplasmic staining observed in adnexal lesions. The staining for PRAME in BCCs and SCCs was heterogeneous, with variable staining intensity among different tumor cells within the same lesion (Table 4 and Supplementary Table S4).

## 4. Discussion

PRAME was first described in 1997 by Ikeda et al. It was recognized as a tumor antigen by human leukocyte antigen (HLA)-A restricted cytotoxic T-lymphocytes [1]. Diffuse PRAME positivity was demonstrated in melanoma, highlighting its utility in characterizing challenging melanocytic neoplasms [15].

The accurate classification of cutaneous neoplasms poses a diagnostic challenge at times, such as distinguishing between sebaceous and non-sebaceous adnexal lesions or differentiating common skin cancers from adnexal carcinomas. In this study, we evaluated staining for PRAME, a tumor-associated antigen, in various types of cutaneous lesions to assess its potential supportive diagnostic utility.

Our findings demonstrate consistent and robust PRAME staining in sebaceous lesions, with cytoplasmic staining observed in sebaceous cells. This distinctive staining pattern, characterized by moderate to strong intensity and peripheral localization within sebaceous units, provides valuable diagnostic clues for supporting the identification of sebaceous neoplasms. Notably, the sensitivity and specificity of PRAME staining for supporting the distinction between sebaceous and non-sebaceous adnexal lesions were 100.0% and 86.7%, respectively, highlighting its potential as a supportive diagnostic marker in this context.

In contrast to sebaceous lesions, PRAME staining was less prevalent in non-sebaceous adnexal lesions, particularly those of hair follicle origin. The absence of PRAME staining in follicular lesions suggests potential diagnostic utility in differentiating these entities from sebaceous lesions. A subset of sweat gland tumors exhibited cytoplasmic PRAME staining, albeit with lower percentages and intensity compared to sebaceous lesions. Further studies are warranted to elucidate the significance of PRAME staining in sweat gland neoplasms and its potential diagnostic and prognostic implications.

Our study also revealed distinct PRAME staining patterns in common skin cancers, namely basal cell carcinomas (BCCs) and squamous cell carcinomas (SCCs). While BCCs and SCCs mostly exhibited weak to moderate nuclear staining for PRAME, sebaceous neoplasms demonstrated cytoplasmic staining, facilitating differentiation between these entities. The heterogeneous staining of PRAME within BCCs and SCCs underscores the importance of careful interpretation and correlation with histopathological features for accurate diagnosis. In a study by Ng et al., the authors showed that the staining pattern of PRAME was predominantly cytoplasmic in normal apocrine glands, germinative sebocytes of sebaceous glands, and hair germs, highlighting its expression in various cutaneous structures. Also, BCCs, SCCs, and sebaceous carcinomas exhibited low levels of PRAME immunoreactivity, with variable proportions of cases showing nuclear staining, similar to melanoma [10].

It has been shown that PRAME is useful in subclassifying sebaceous carcinoma according to the degree of differentiation, where well-differentiated grades 1 and 2 stain strongly for PRAME whereas it is almost completely absent in grade 3 [16,17,18]. Future studies exploring the molecular mechanisms underlying PRAME staining in sebaceous neoplasms may provide valuable insights into their pathogenesis and potential therapeutic targets.

Limitations of this study include its retrospective design, which may introduce selection bias, and the inherent variability in immunohistochemical staining interpretation. Additionally, the relatively small sample size for certain subgroups of cutaneous neoplasms may limit the generalizability of our findings. Future prospective studies with larger cohorts are warranted to validate our observations and further elucidate the diagnostic utility of PRAME in cutaneous pathology.

## 5. Conclusions

In conclusion, our study is amongst the first studies to highlight the potential diagnostic utility of PRAME immunohistochemistry in supporting the distinction between sebaceous and non-sebaceous adnexal lesions. The distinctive PRAME staining patterns observed in various cutaneous tumors underscore its potential clinical implications for guiding diagnostic and therapeutic strategies. Further research elucidating the biological significance of PRAME staining in cutaneous neoplasms is warranted to optimize its utility in clinical practice.

## Figures and Tables

**Table 1:** PRAME cytoplasmic positivity in sebaceous vs. non-sebaceous adnexal lesions.

	Sebaceous (*n* = 17)	Non-Sebaceous (*n* = 45)	*p*-Value
PRAME Positivity			<0.001
Negative	0 (0.0%)	39 (86.7%)	
Positive	17 (100.0%)	6 (13.3%)	

**Table 2:** PRAME % in sebaceous vs. non-sebaceous adnexal lesions.

	Sebaceous (*n* = 17)	Non-Sebaceous (*n* = 45)	*p*-Value
PRAME %			<0.001
0%	0 (0.0%)	39 (86.7%)	
1–25%	0 (0.0%)	1 (2.2%)	
26–50%	2 (11.8%)	0 (0.0%)	
51–75%	2 (11.8%)	2 (4.4%)	
>75%	13 (76.4%)	3 (6.7%)	

**Table 3:** PRAME intensity in sebaceous vs. non-sebaceous adnexal lesions.

	Sebaceous (*n* = 17)	Non-Sebaceous (*n* = 45)	*p*-Value
PRAME Intensity			<0.001
Negative	0 (0.0%)	39 (86.7%)	
Weak	5 (29.4%)	1 (2.2%)	
Moderate	3 (17.6%)	2 (4.4%)	
Severe	9 (53.0%)	3 (6.7%)	

**Table 4:** PRAME nuclear positivity in BCC and SCC.

	BCC (*n* = 10)	SCC (*n* = 9)
PRAME Positivity		
Negative	0 (0.0%)	2 (22.2%)
Positive	10 (100.0%)	7 (77.8%)

## Data Availability

No new data were created or analyzed in this study. The original contributions presented in the study are included in the article/Supplementary Materials, further inquiries can be directed to the corresponding author.

## Supplementary Materials

The following supporting information can be downloaded at: https://www.mdpi.com/article/10.3390/dermatopathology11040039/s1, Supplementary Table S1. PRAME intensity and % in different subtypes of sebaceous lesions; Supplementary Table S2. PRAME intensity and % in different subtypes of follicular lesions; Supplementary Table S3. PRAME intensity and % in different subtypes of sweat gland lesions; Supplementary Table S4. PRAME intensity and % in squamous cell carcinoma and basal cell carcinoma.
