# Supplementary material for: PRAME Staining of Adnexal Lesions and Common Skin Cancer Types: Biomarker with Potential Diagnostic Utility
Source: Dermatopathology (Basel). 2024 Dec 12;11(4):364–73. doi: 10.3390/dermatopathology11040039 (PMC11674263; doi:10.3390/dermatopathology11040039)
Supplement: Supplementary file 1 [file dermatopathology-11-00039-s001.zip › Supplementary Table 1.pdf]

**Supplementary Table 1.** PRAME intensity and % in different subtypes of sebaceous lesions.

| Case | Lesion | Intensity | % |
|---|---|---|---|
| **BENIGN SEBACEOUS LESIONS** | | | |
| **Case 1** | Prominent benign sebaceous glands | Weak | 76-100% |
| **Case 2** | Prominent benign sebaceous glands | Weak | 76-100% |
| **Case 3** | Prominent benign sebaceous glands | Strong | 76-100% |
| **Case 4** | Prominent benign sebaceous glands | Strong | 76-100% |
| **Case 5** | Sebaceous hyperplasia | Strong | 76-100% |
| **Case 6** | Heterotopic / ectopic sebaceous glands | Moderate | 76-100% |
| **Case 7** | Heterotopic / ectopic sebaceous glands | Moderate | 26-50% |
| **Case 8** | Heterotopic / ectopic sebaceous glands | Strong | 76-100% |
| **Case 9** | Heterotopic / ectopic sebaceous glands | Strong | 76-100% |
| **Case 10** | Sebaceous adenoma | Strong | 76-100% |
| **Case 11** | Sebaceous adenoma | Strong | 76-100% |
| **Case 12** | Sebaceous epithelioma | Strong | 51-75% |
| **Case 13** | Steatocystoma | Strong | 76-100% |
| **MALIGNANT SEBACEOUS NEOPLASMS** | | | |
| **Case 14** | Sebaceous carcinoma, well differentiated | Weak | 76-100% |
| **Case 15** | Sebaceous carcinoma, well differentiated | Moderate | 76-100% |
| **Case 16** | Sebaceous carcinoma, moderately differentiated | Weak | 51-75% |
| **Case 17** | Sebaceous carcinoma, poorly differentiated | Weak | 26-50% |
