# Supplementary material for: PRAME Staining of Adnexal Lesions and Common Skin Cancer Types: Biomarker with Potential Diagnostic Utility
Source: Dermatopathology (Basel). 2024 Dec 12;11(4):364–73. doi: 10.3390/dermatopathology11040039 (PMC11674263; doi:10.3390/dermatopathology11040039)
Supplement: Supplementary file 1 [file dermatopathology-11-00039-s001.zip › Supplementary Table 2.pdf]

Supplementary Table 2. PRAME intensity and % in different subtypes of follicular lesions.

Case	Lesion	Intensity	%
**BENIGN FOLLICULAR LESIONS**			
**Case 1**	Trichoadenoma	0	0%
**Case 2**	Pilomatricoma	0	0%
**Case 3**	Pilomatricoma	0	0%
**Case 4**	Pilomatricoma	0	0%
**Case 5**	Pilomatricoma	0	0%
**Case 6**	Pilomatricoma	0	0%
**Case 7**	Pilomatricoma	0	0%
**Case 8**	Pilomatricoma	0	0%
**Case 9**	Pilomatricoma	0	0%
**Case 10**	Pilomatricoma	0	0%
**Case 11**	Pilomatricoma	0	0%
**Case 12**	Proliferating Trichilemmal cyst	0	0%
**Case 13**	Proliferating Trichilemmal cyst	0	0%
