# Supplementary material for: PRAME Staining of Adnexal Lesions and Common Skin Cancer Types: Biomarker with Potential Diagnostic Utility
Source: Dermatopathology (Basel). 2024 Dec 12;11(4):364–73. doi: 10.3390/dermatopathology11040039 (PMC11674263; doi:10.3390/dermatopathology11040039)
Supplement: Supplementary file 1 [file dermatopathology-11-00039-s001.zip › Supplementary Table 3.pdf]

Supplementary Table 3. PRAME intensity and % in different subtypes of sweat gland lesions.

Case	Lesion	Intensity	%
**BENIGN SWEAT GLAND LESIONS**			
**Case 1**	Apocrine and eccrine gland hyperplasia	Strong	76-100%
**Case 2**	Apocrine hidrocystoma	Strong	51-75%
**Case 3**	Apocrine hidrocystoma	0	0%
**Case 4**	Eccrine hidrocystoma	0	0%
**Case 5**	Eccrine poroma	0	0%
**Case 6**	Eccrine poroma	0	0%
**Case 7**	Eccrine poroma	0	0%
**Case 8**	Eccrine poroma	0	0%
**Case 9**	Eccrine spiradenoma	0	0%
**Case 10**	Eccrine spiradenoma	0	0%
**Case 11**	Eccrine spiradenoma	0	0%
**Case 12**	Eccrine spiradenoma	0	0%
**Case 13**	Eccrine syringosquamous metaplasia	0	0%
**Case 14**	Hidradenoma papilliferum	0	0%
**Case 15**	Hidradenoma papilliferum	0	0%
**Case 16**	Hidradenoma papilliferum	Strong	1-25%
**Case 17**	Hidrocystoma	0	0%
**Case 18**	Hidrocystoma	0	0%
**Case 19**	Hidrocystoma	0	0%
**Case 20**	Hidrocystoma	Weak	51-75%
**Case 21**	Hidrocystoma	Moderate	76-100%
**Case 22**	Papillary hidradenoma	Moderate	76-100%
**Case 23**	Poroid hidradenoma	0	0%
**Case 24**	Syringoma	0	0%
**Case 25**	Syringoma	0	0%
**Case 26**	Syringoma	0	0%

MALIGNANT SWEAT GLAND NEOPLASMS			
**Case 27**	Eccrine carcinoma	0	0%
**Case 28**	Eccrine carcinoma	0	0%
**Case 29**	Eccrine carcinoma	0	0%
**Case 30**	Metastatic eccrine carcinoma	0	0%
**Case 31**	Porocarcinoma	0	0%
**Case 32**	Clear cell hidradenocarcinoma	0	0%
