# Supplementary material for: PRAME Staining of Adnexal Lesions and Common Skin Cancer Types: Biomarker with Potential Diagnostic Utility
Source: Dermatopathology (Basel). 2024 Dec 12;11(4):364–73. doi: 10.3390/dermatopathology11040039 (PMC11674263; doi:10.3390/dermatopathology11040039)
Supplement: Supplementary file 1 [file dermatopathology-11-00039-s001.zip › Supplementary Table 4.pdf]

Supplementary Table 4. PRAME intensity and % in squamous cell carcinoma and basal cell carcinoma.

Case	Intensity	%
**Squamous cell carcinoma**		
**Case 1**	Weak	76-100%
**Case 2**	Weak	76-100%
**Case 3**	Weak	76-100%
**Case 4**	Weak	51-75%
**Case 5**	Weak	51-75%
**Case 6**	Weak	51-75%
**Case 7**	Weak	51-75%
**Case 8**	0	0%
**Case 9**	0	0%
**Basal cell carcinoma**		
**Case 1**	Weak	76-100%
**Case 2**	Weak	76-100%
**Case 3**	Weak	51-75%
**Case 4**	Moderate	76-100%
**Case 5**	Moderate	76-100%
**Case 6**	Moderate	76-100%
**Case 7**	Moderate	76-100%
**Case 8**	Strong	76-100%
**Case 9**	Strong	76-100%
**Case 10**	Strong	76-100%
